# Alcohol Related Brain Damage Presentations in an Acute General Hospital

**DOI:** 10.1192/bjo.2022.266

**Published:** 2022-06-20

**Authors:** Talha Muneer Amanullah, David Henstock, Bushra Azam

**Affiliations:** Derbyshire Healthcare NHS Foundation Trust, Chesterfield, United Kingdom

## Abstract

**Aims:**

Alcohol-related brain damage (ARBD) is used to describe a variety of clinical syndromes associated with excessive intake of alcohol. It can present with cognitive and neurological syndromes, including Wernicke's encephalopathy, Korsakoff's syndrome, alcohol dementia, cerebellar atrophy and frontal lobe dysfunction, Central pontine myelinolysis and Marchiafava Bignami disease. In up to 25% of cases ARBD can be complicated by traumatic head injury and brain blood supply disturbances. In the absence of clear national guidelines, standards or established pathways of care across most of the UK, most patients are unable to access appropriate service provision. The North Derbyshire mental health liaison team (MHLT) provides assessment and diagnosis of acute alcohol related brain injury, assess severity (based on clinical presentation, investigation findings, cognitive assessment) and provide a care plan with follow-up to various community services. Aim and objectives: To find out the discharge outcome for patients with ARBD diagnosis by the north MHLT, help us identify service gaps and look at ways to improve patient's care in this group.

**Methods:**

We retrospectively analysed 300 patients who were referred to liaison team for drug and alcohol problems and were seen by the drug and alcohol lead nurse within the liaison team. Patients who were given a diagnosis of ARBD by the liaison team were included in the study.

We looked at

1. Age and gender distribution
2. Team who gave the initial diagnosis
3. Discharge destination
4. Community follow-up and engagement

**Results:**

We identified 17 patients who were given diagnosis of ARBD. There was relatively equal distribution of male to female patients. Majority of diagnosis’ were given by liaison team. The discharge destination was variable with around half referred to ARBD rehabilitation unit and Derbyshire recovery partnership. Engagement was poor with only 20% of patients engaging with services.

**Conclusion:**

Recommendations:

1. Detailed cognitive tests need doing for screening and to establish severity

2. Consideration for which neuroimaging modalities can help aid diagnosis, if any, should be made.

1. ARBD leaflets to be given
2. ARBD diagnosed patients who do not need rehabilitation unit, should be referred for social care assessment as an inpatient and / or be followed up in the community under Care Act

5. Considerations with the Multi Disciplinary Team for ways to improve engagement in the community, perhaps with more frequent and robust follow-ups.

